# Microbial phenolic metabolites at the neurovascular interface: a precision-nutrition framework for brain aging

**DOI:** 10.3389/fnut.2026.1895218

**Published:** 2026-08-18

**Authors:** Sayandeep K. Das, Mamatha K, Savitri M. Nerune, Afsona Parveen, Prithviraj Karak

**Affiliations:** 1 Department of Pathology, Shri B. M. Patil Medical College Hospital and Research Centre, BLDE (Deemed to be University), Vijayapura, Karnataka, India; 2 Department of Bachelor in Medical Laboratory Technology, Durgapur Institute of Paramedical Science, Durgapur, West Bengal, India; 3 Department of Physiology, Bankura Christian College, Bankura, India

**Keywords:** blood–brain barrier, brain aging, dietary polyphenols, gut microbiota, microbial phenolic metabolites, neuroinflammation, neurovascular unit, precision nutrition

## Abstract

Brain aging is increasingly recognized as a neurovascular and inflammatory process involving blood–brain barrier dysfunction, endothelial activation, pericyte impairment, astrocyte–microglia reactivity, oxidative stress, white matter vulnerability, and cognitive decline. Although polyphenol-rich diets are widely associated with brain-health benefits, many parent polyphenols have limited systemic bioavailability, suggesting that gut microbiota-derived microbial phenolic metabolites may represent more biologically relevant circulating exposures. However, systemic MPM exposure is also conditioned by intestinal barrier integrity, which may change with aging and determine the magnitude, timing, and inflammatory context of metabolite entry into the circulation. This Mini Review examines microbial phenolic metabolites as proximate mediators at the blood–brain barrier and neurovascular unit, rather than as indirect extensions of parent dietary polyphenols. We synthesize human and translational evidence linking urinary and plasma microbial phenolic metabolites, including phenyl-*γ*-valerolactones, urolithins, enterolignans, phenolic acids, and conjugated metabolites, with exposure assessment, brain-adjacent detection, and cognitive aging phenotypes. We also review mechanistic evidence showing that low-molecular-weight phenolic metabolites can cross blood–brain barrier models, modify endothelial barrier integrity, influence tight-junction and adherens-junction organization, and attenuate inflammatory signaling in endothelial and microglial systems. Building on this evidence, we propose a hypothesis-generating gut-barrier–MPM–BBB/NVU response modelmodel in which diet-derived MPMs may be linked to brain-aging resilience through candidate effects on blood–brain barrier preservation, endothelial inflammatory restraint, pericyte and glial support, and downstream cognitive outcomes. Finally, we outline a pathology-informed precision-nutrition framework integrating dietary assessment, urine/plasma metabolomics, fecal microbial enzyme capacity, intestinal permeability markers, neurovascular biomarkers, imaging readouts, and cognitive testing to identify potential neurovascular responder metabotypes.

## 1 Introduction: why brain aging needs a neurovascular-metabolite lens

Brain aging is increasingly understood as a disorder of neurovascular resilience, in which vascular, glial, inflammatory, and metabolic stressors progressively erode brain homeostasis (1). BBB dysfunction is increasingly viewed not simply as a late epiphenomenon, but as a clinically relevant feature of aging and neurodegeneration, with human imaging studies demonstrating age-related BBB breakdown in the hippocampus and related medial temporal lobe structures (1, 2). The BBB is embedded within the neurovascular unit (NVU), a multicellular interface comprising endothelial cells, tight and adherens junctions, pericytes, vascular smooth muscle cells, basement membrane, astrocytic endfeet, neurons, microglia, and extracellular matrix (1). Because circulating microbial phenolic metabolites (MPM) encounter the cerebral endothelium before they encounter neurons, the BBB/NVU should be evaluated as a candidate primary interface for diet-derived metabolite action (3).

Polyphenol-rich diets are frequently framed through antioxidant or parent-compound mechanisms, but this view is biologically incomplete because many dietary polyphenols undergo extensive gut microbial and host metabolism before reaching systemic circulation (3, 4). Low-molecular-weight MPMs generated from dietary polyphenols may modulate BBB homeostasis by influencing junctional proteins, cytokine secretion, and oxidative stress (3). Accordingly, brain-aging research may benefit from shifting beyond reported polyphenol intake alone toward measured microbial phenolic metabolites (3). This matrix-specific approach is important because urine captures cumulative microbial-metabolic signatures, whereas plasma/serum better represents the circulating BBB-facing metabolite pool that may interact with endothelial and glial components (3).

An upstream barrier layer should also be incorporated into this framework. The BBB-centered model should not be read as starting only at the cerebral endothelium; circulating MPM availability is first shaped by intestinal epithelial barrier integrity, mucus and tight-junction function, microbial enzyme activity, epithelial uptake, phase-II conjugation, enterohepatic handling, and renal clearance. Increased intestinal permeability, commonly described as “leaky gut,” may become more relevant during aging and may alter both the concentration–time profile of MPMs and the inflammatory milieu in which they reach the BBB/NVU. Polyphenols may therefore influence neurovascular exposure through two linked mechanisms: by serving as substrates for microbial conversion and by modulating gut barrier function and gut microbial ecology. This creates a multi-layered barrier cascade: dietary substrate → gut microbial biotransformation → intestinal barrier passage → circulating MPM profile → BBB/NVU response (5–7).

**Figure 1:**
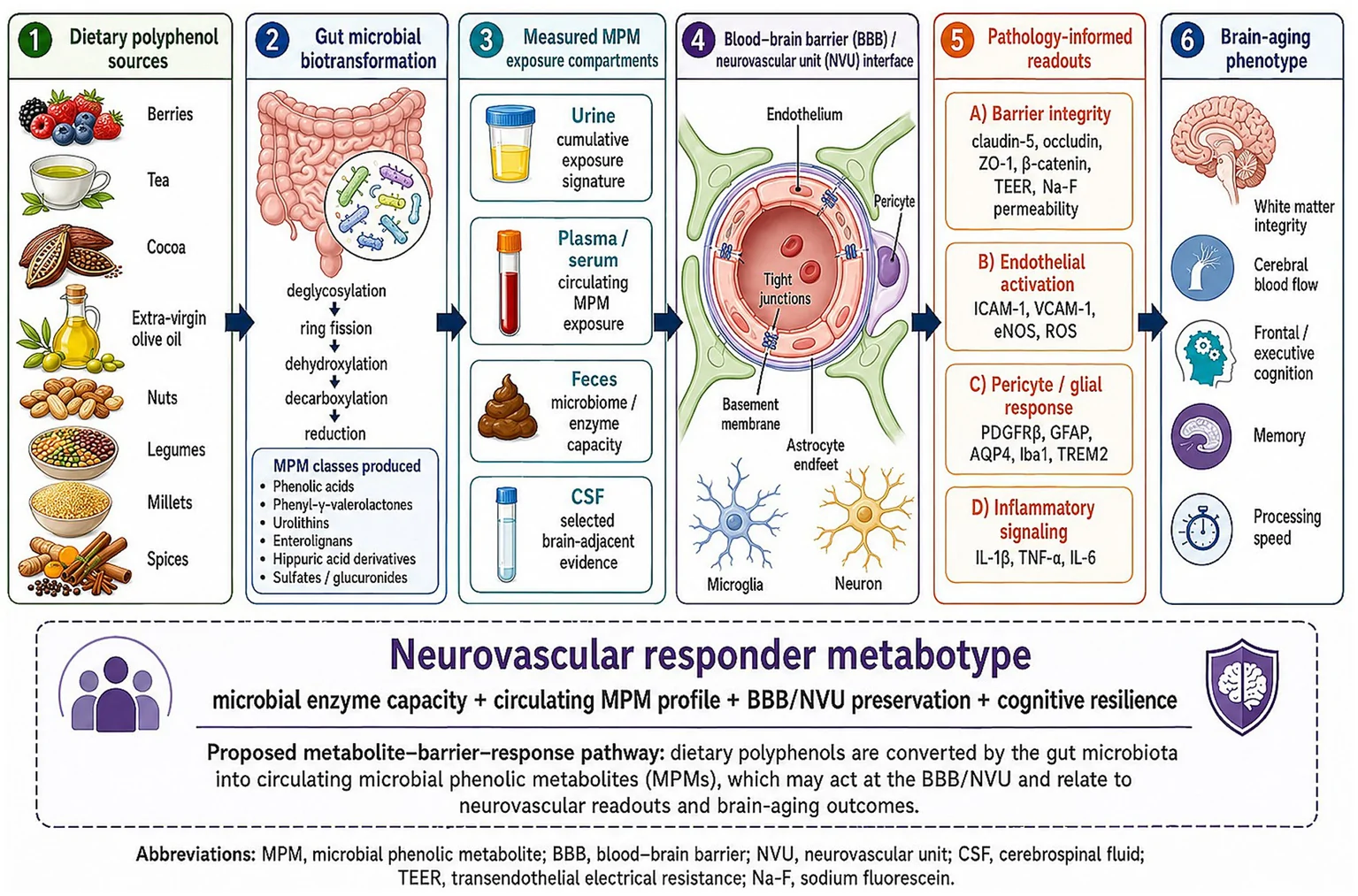
Proposed metabolite–barrier–response framework linking dietary polyphenols, gut microbial biotransformation, microbial phenolic metabolites, and neurovascular brain aging. Parent dietary polyphenols undergo microbial conversion to smaller circulating MPMs, including phenolic acids, phenyl-*γ*-valerolactones, urolithins, enterolignans, and sulfate/glucuronide conjugates. Urinary MPMs provide cumulative exposure signatures, whereas plasma/serum metabolites represent the BBB-facing circulating pool. At the neurovascular interface, candidate responses include BBB junctional integrity, endothelial activation, pericyte support, astrocyte–microglia inflammatory tone, white-matter vulnerability, and frontal/executive cognitive outcomes. This framework is hypothesis-generating and does not establish causality.

A pathology-informed nutrition framework also requires tissue-level readouts rather than reliance on dietary questionnaires and cognitive scores alone (1, 3). Candidate NVU readouts include claudin-5, occludin, ZO-1, albumin/IgG leakage, ICAM-1, VCAM-1, eNOS, PDGFRβ, pericyte coverage, GFAP, AQP4 polarization, Iba1, TREM2, IL-1β, TNF-*α*, white matter integrity, and frontal/executive cognition (1, 3). Recent microbiome mapping strengthens this precision-nutrition perspective by linking 775 edible-plant phytonutrients to microbial enzymes across 3,068 human gut microbiomes, with marked interpersonal and geographic variability in biotransformation potential (4). This Mini Review therefore examines MPMs as candidate proximate neurovascular exposureand proposes a metabolite–barrier–response model for identifying neurovascular responder metabotypes in brain aging (Figure 1) (3, 4).

### 1.1 Literature identification and evidence selection

This Mini Review was developed through a targeted narrative literature search performed in PubMed/MEDLINE, Scopus, Web of Science, and Google Scholar from database inception to June 2026. Search terms combined concepts related to dietary polyphenols and microbial metabolism (“polyphenol,” “microbial phenolic metabolite,” “phenyl-*γ*-valerolactone,” “urolithin,” “enterolignan,” “phenolic acid,” “glucuronide,” “sulfate”) with neurovascular and brain-aging terms (“blood–brain barrier,” “neurovascular unit,” “brain aging,” “cognition,” “mild cognitive impairment,” “dementia,” “neuroinflammation,” and “oxidative stress”). We prioritized peer-reviewed human studies measuring urinary, plasma, serum, or cerebrospinal-fluid metabolites; translational studies examining BBB or neurovascular-unit access; and experimental studies reporting barrier integrity, endothelial activation, glial inflammatory signaling, or cognition-relevant outcomes. Studies focused only on parent polyphenol intake without metabolite, microbiome, BBB/NVU, or cognition-relevant endpoints were used only for background context. Reference lists of key reviews and primary studies were manually screened to identify additional relevant articles.

## 2 From dietary polyphenols to microbial phenolic metabolites

Because many MPMs arise from colonic microbial metabolism, the intestine should be treated as both the site of production and the first barrier determining systemic exposure. Increased intestinal permeability in older adults may increase host exposure to microbial products and inflammatory mediators while also modifying MPM bioavailability. In the MaPLE randomized crossover trial, a polyphenol-rich dietary pattern reduced serum zonulin, a surrogate marker of intestinal permeability, in older adults with increased permeability. Subsequent multi-omics analysis linked polyphenol-related serum metabolites, butyrate-producing taxa, and zonulin changes, supporting a gut barrier–microbiota–metabolome interaction. These findings suggest that polyphenols may shape systemic MPM exposure not only by supplying microbial substrates but also by modifying the upstream intestinal barrier. Future MPM studies should therefore measure intestinal permeability markers, such as serum zonulin, lactulose/mannitol ratio, intestinal fatty-acid binding protein, LBP/sCD14, endotoxin/LPS, and fecal calprotectin, alongside urine and plasma MPM profiles (5–7).

Polyphenol-rich foodsincluding berries, tea, cocoa, extra-virgin olive oil, nuts, legumes, millets and spicessupply diverse flavonoids, phenolic acids, stilbenes, lignans and tannins, but dietary availability should not be equated with systemic neurovascular exposure (8, 9). Many parent polyphenols are poorly absorbed in the small intestine or rapidly conjugated, leaving a substantial fraction to reach the colon where microbial enzymes perform deglycosylation, ring fission, dehydroxylation, decarboxylation and reduction reactions (9–11). This conversion generates smaller and more absorbable microbial phenolic metabolites, including phenolic acids, phenyl-*γ*-valerolactones, urolithins, enterolignans, hippuric acid derivatives, and sulfate or glucuronide conjugates (10–14). Dietary intake should therefore be considered the input, microbial biotransformation the exposure-generating step, and circulating phenolic metabolites the candidate effectors (4, 12–14).

The biomarker literature is especially instructive because it shows that food-frequency estimates can be strengthened by measured metabolite exposure (12, 13). Ottaviani et al. developed a validated high-throughput urinary assay for microbiome-derived 5-(3′,4′-dihydroxyphenyl)-*γ*-valerolactone metabolites and reported a strong relationship between flavan-3-ol intake and biomarker concentration, supporting *γ*-valerolactone metabolites as objective exposure markers for tea-, fruit-, wine- and cocoa-derived flavan-3-ols (12). Parmenter et al. subsequently showed that urinary phenyl-γ-valerolactones can function as dietary flavan-3-ol biomarkers, with two principal PVLs accounting for more than 75% of excreted PVLs and the summed principal PVLs correlating with intake (13). Angelino et al. extended this exposure logic to blood by showing that plasma PVL1 plus PVL2 increased across flavan-3-ol intake quartiles in older adults, indicating that microbial catabolites can be quantified in circulation (14).

Biological matrices should therefore be selected according to the mechanistic question rather than convenience alone (4, 12–15). Urine is suited for cumulative exposure and microbial-metabolic signatures, whereas plasma or serum is more informative for estimating the circulating, BBB-facing metabolite pool that may interact with the neurovascular unit (4, 12–15). Fecal samples add a complementary layer by enabling microbiome composition, metagenomic enzyme capacity and meta transcriptomic activity to be linked with metabolite output (4). CSF is not practical for routine nutrition studies, but in selected clinical or translational cohorts it can provide evidence that circulating (poly)phenol-derived metabolites or related methylxanthine metabolites access brain-adjacent fluid compartments (15). This matrix-specific approach is essential because individuals differ substantially in microbial biotransformation capacity, producing responder and non-responder metabotypes for metabolites such as urolithins, enterolignans and valerolactones (4, 10). Recent large-scale microbiome mapping strengthened this precision-nutrition argument by linking 775 edible-plant phytonutrients to microbial enzymes across 3,068 global human gut microbiomes, demonstrating marked interpersonal and geographical variability in phytonutrient transformation potential (4).

## 3 The neurovascular unit as the missing target

The neurovascular unit (NVU) provides a pathology-informed framework for studying circulating MPMs because it integrates the vascular, glial and neuronal compartments that regulate brain homeostasis and is increasingly implicated in neurodegenerative vulnerability (1, 3, 16). Rather than treating the gut–brain axis as a diffuse signaling pathway, an NVU framework focuses on the BBB as the first brain-facing interface for blood-borne metabolites and asks whether metabolite exposure is associated with, or experimentally modifies, barrier integrity (3, 17). Endothelial barrier integrity should be assessed through claudin-5, occludin, ZO-1 and *β*-catenin localization, together with functional readouts such as transendothelial electrical resistance (TEER), sodium fluorescein (Na-F) permeability, Evans blue extravasation and albumin/IgG leakage (1, 18, 19). Endothelial activation should be evaluated through ICAM-1, VCAM-1, eNOS and ROS-related markers, while pericyte and basement-membrane support should be captured by PDGFRβ signaling, pericyte coverage and matrix stability (1, 3). Astrocytic and microglial responses should include GFAP, AQP4 polarization, Iba1, CD68, TREM2, IL-1β and TNF-*α*, because these readouts indicate whether metabolite exposure modifies endothelial–glial inflammatory circuits rather than only neuronal survival (1, 17, 19).

The most informative mechanistic studies are not those showing that a compound is “neuroprotective” in neurons, but those linking physiologically plausible metabolite concentrations to BBB transport, barrier phenotype, endothelial inflammation and glial response (3, 17, 19). Figueira et al. showed that berry-derived phenolic sulfates tested at 5 μM crossed an HBMEC BBB model and that pyrogallol sulfate reduced LPS-induced microglial TNF-*α* release by approximately two-fold through NF-κB/IκBα modulation (17). Angelino et al. extended this principle to flavan-3-ol microbial catabolites by showing that 5-(hydroxyphenyl)-*γ*-valerolactone-sulfate crossed BBB-relevant models after 5 μM exposure for 2 h and was detected in rat and pig brain tissues (14). Carecho et al. moved beyond permeability alone by showing that pyrogallol sulfate had high BBB permeability, improved *β*-catenin membrane expression and reduced ZO-1 membrane gaps, while injected metabolites reached rat brain rapidly (18). Garay-Mayol et al. strengthened the urolithin arm of this model by showing that urolithins and phase-II conjugates crossed HBMEC BBB models, Uro-A reduced TNFα-induced Na-F permeability, selected metabolites rescued TEER, and urolithins reduced LPS-induced microglial IL-6 and NF-κB nuclear translocation (19). Thus, BBB transport is necessary but insufficient; neurovascular relevance requires coupling metabolite access to barrier preservation, cytokine regulation, glial restraint and ultimately cognitive or neuroimaging phenotypes (3, 18, 19).

## 4 Human and translational evidence: promising, but not yet causal

Human evidence now supports testing a metabolite-centered hypothesis for diet-derived neuroprotection, but the current evidence base remains stronger for exposure characterization and association than for causal neurovascular modification (13–15, 20). The most direct cognitive evidence comes from Domínguez-López et al., who reported that urinary MPMs were associated with better frontal-lobe function in older PREDIMED participants (20). This study is important because it links measured MPM exposure with executive/frontal cognitive performance, a domain highly relevant to vascular aging, but its cross-sectional design prevents causal inference (20). Thus, urinary MPMs should be interpreted as candidate neurocognitive exposure biomarkers rather than proof that microbial metabolites directly preserve brain function (20).

Brain-access plausibility is strengthened by Le Sayec et al., who analyzed paired plasma and cerebrospinal fluid from individuals at risk of dementia and showed that multiple diet-derived phenolic and methylxanthine metabolites were detectable in CSF (15). These data support the possibility that circulating dietary metabolites can reach brain-adjacent compartments, but they do not establish whether such exposure modifies BBB integrity, neurovascular signaling, or cognition (15). This distinction is central to the present review: detection in plasma or CSF is biologically informative, but neurovascular relevance requires linkage to barrier phenotype, endothelial inflammation, glial response, imaging, or cognitive outcomes (15).

Flavan-3-ol biomarker studies provide a second translational pillar by showing that microbial catabolites can objectively capture dietary exposure (12–14). Angelino et al. showed that plasma phenyl-*γ*-valerolactones increased with habitual flavan-3-ol intake in older adults, supporting PVLs as circulating, BBB-facing exposure markers (14). Parmenter et al. validated urinary PVLs as exposure biomarkers, while Ottaviani et al. demonstrated that microbiome-derived γ-valerolactone metabolites can be used at scale to estimate flavan-3-ol intake more objectively than food-frequency inference alone (12, 13). Together, these studies support incorporating measured metabolite exposure alongside parent polyphenol intake (12–14).

Intervention trials show why metabolite-resolved interpretation is necessary (21–23). Earlier cocoa-flavanol trials reported improvements in dentate-gyrus-related function or executive-performance measures in older adults, but they generally did not measure microbial metabolite exposure, microbiome conversion capacity, or BBB/NVU biomarkers (21, 22). In contrast, the COSMOS-Clinic sub cohort found no overall two-year cognitive benefit from cocoa extract supplementation, making it an essential cautionary study against broad claims that polyphenol intake alone is neuroprotective (23). These mixed findings suggest that cognitive responses to polyphenol-rich interventions may depend on baseline diet quality, microbial conversion capacity, circulating metabolite profiles, vascular risk, and neurovascular vulnerability (13, 14, 21–23). Human studies therefore increasingly support MPMs as exposure biomarkers and candidate neurocognitive correlates, but causal trials that simultaneously measure diet, microbial enzyme capacity, circulating metabolites, BBB/NVU response, and cognition remain scarce (Table 1).

**Table 1:** Human evidence domains linking MPMs with neurocognitive aging.

Evidence domain	Key study/studies	Study design/study population	Quantitative signal	Translational implication
Direct MPM–cognition association	Domínguez-López et al. (20)	Cross-sectional PREDIMED-Plus older-adult study	*n* = 400PREDIMED participants; urinary MPM score associated with better frontal-lobe function; VAG and UBG linked to better CTT-2 performance	Strongest direct human evidence that urinary MPMs may mark frontal/executive cognitive resilience; cross-sectional, so causality remains unresolved
Human brain-access plausibility	Le Sayec et al. (15)	Observational paired plasma/CSF biofluid study	*n* = 90 at risk of dementia; 123 metabolites in CSF, 127 in plasma; 5/20 quantified CSF metabolites predicted to cross by passive diffusion, ≥9 likely transporter-assisted	Supports the biological plausibility that diet-derived metabolites can reach brain-adjacent compartments; does not prove NVU function or cognitive benefit
Circulating flavan-3-ol catabolites	Angelino et al. (14)	Older-adult cohort biomarker association study	Older-adult dietary subset *n* = 557; plasma PVL1 + PVL2 increased from 28.3 nmol/L in Q1 to 45.2 nmol/L in Q4 of flavan-3-ol intake	Shows that microbial PVLs are measurable in blood and can represent BBB-facing exposure; no direct cognition or BBB endpoint
Urinary exposure biomarkers	Ottaviani et al. (12) Parmenter et al. (13)	Biomarker-validation studies; crossover RCT plus observational validation	Parmenter: 2 PVLs >75% of excreted PVLs; summed PVLs correlated with intake, Rs = 0.37, *p* = 0.0004. Ottaviani: urinary gVLM strongly correlated with flavan-3-ol intake, *ρ* = 0.90	Validates objective metabolite-based exposure assessment; biomarker validity alone does not establish neuroprotection
Positive cognitive/imaging trial signal	Brickman et al. (21) Mastroiacovo et al. (22)	Randomized flavanol intervention trials	Brickman: high-flavanol intervention enhanced dentate-gyrus function by fMRI and cognitive testing. Mastroiacovo: cocoa flavanol doses 993/520/48 mg/day, with strongest TMT/VFT improvements in the high-flavanol group	Shows cognitive relevance of flavanol-rich exposure, but microbial metabolites, microbiome capacity, and BBB/NVU markers were not measured
Neutral/balancing RCT evidence	COSMOS-Clinic Vyas et al. (23)	Two-year randomized cocoa-extract sub cohort	*n* = 573; cocoa extract 500 mg/day flavanols, including 80 mg/day epicatechin; no overall 2-year benefit: global cognition −0.01 SU, episodic memory −0.01 SU, executive function/attention 0.003 SU	Prevents overclaiming; supports the need for metabolite-resolved and metabotype-stratified trials

Overall, the human evidence should be interpreted as hypothesis-generating rather than causal. Most available studies either characterize exposure biomarkers or report associations between measured metabolites and cognitive or brain-adjacent phenotypes. Cross-sectional designs cannot determine temporal directionality, and intervention trials have rarely measured diet, microbial enzyme capacity, circulating MPMs, BBB/NVU biomarkers, neuroimaging, and cognition within the same participants. Therefore, current human data support MPMs as promising exposure biomarkers and candidate neurocognitive correlates, but they do not yet establish that MPMs directly preserve BBB integrity or prevent cognitive decline.

## 5 Mechanistic BBB/NVU evidence: transport, barrier phenotype, and inflammatory response

Mechanistic evidence for MPMs is strongest when organized as a layered NVU sequence: BBB access, barrier-phenotype modulation, neuroimmune signaling, and pathology-state validation (17–19, 24–27). The first layer is transport, because circulating metabolites must reach or interact with the cerebral endothelial interface before they can plausibly influence brain aging (17–19, 24). Figueira et al. showed that berry-derived phenolic sulfates tested at physiologically relevant low-micromolar concentrations crossed an HBMEC BBB model and produced changes consistent with protection in neuronal and microglial systems (17). Angelino et al. extended this transport evidence to flavan-3-ol microbial catabolites by showing that 5-(hydroxyphenyl)-*γ*-valerolactonesulfate crossed BBB-relevant models and was detectable in rat and pig brain tissues after dietary exposure (24). Carecho et al. further demonstrated *in vitro* BBB transport and rapid rat brain distribution of selected low-molecular-weight phenolic sulfates, while Garay-Mayol et al. showed that urolithins and their glucuronide or sulfate conjugates crossed HBMEC BBB models (18, 19).

### 5.1 Transporter mechanisms: net permeability is not transporter attribution

Current BBB studies provide evidence of net MPM translocation, but they rarely define the molecular route. Candidate mechanisms include passive transcellular diffusion for smaller and less polar metabolites; carrier-mediated uptake by monocarboxylate transporters, especially MCT1/MCT4, for phenolic acids and other monocarboxylates; OATP/OAT-family transport of anionic sulfate and glucuronide conjugates; vesicular or caveolae-associated pathways; and restriction or efflux by ABC transporters such as BCRP, P-gp, and MRPs. Therefore, phenolic acids, phenyl-*γ*-valerolactonesulfates/glucuronides, and urolithin conjugates should not be assumed to share a single BBB route. Aging, diabetes, neuroinflammation, and neurodegeneration may further modify transporter expression, polarization, and activity, making MPM permeability pathology-dependent. Future BBB/NVU studies should report bidirectional apparent permeability, efflux ratios, transporter inhibitor data, transporter knockdown or overexpression, and proteomic quantification of OATP, MCT, BCRP, P-gp, and MRP proteins in the same BBB model used for metabolite transport (28–31).

The second layer is barrier phenotype, because BBB transport alone does not establish neurovascular protection (18, 19). Carecho et al. linked pyrogallol sulfate exposure to improved endothelial junctional organization, including increased *β*-catenin membrane expression and reduced ZO-1 membrane gaps, thereby moving the field beyond simple permeability assays (18). Garay-Mayol et al. modeled inflammatory BBB disruption with TNFα and showed that Uro-A reduced Na-F permeability, while Uro-A, IsoUro-A and IsoUro-A-3-glucuronide counteracted TEER loss (19). These findings suggest that MPMs and their phase-II conjugates can influence endothelial integrity under inflammatory stress, but this interpretation still requires confirmation in chronic aging models (18, 19).

The third layer is neuroimmune signaling, which connects endothelial exposure to glial inflammatory response (17, 19, 25). Figueira et al. showed that pyrogallol sulfate reduced LPS-induced microglial TNF-*α* release and modulated NF-κB/IκBα signaling, supporting an endothelial–microglial mechanism rather than an isolated neuronal antioxidant effect (17). Garay-Mayol et al. similarly reported stimulus-dependent urolithin effects in HMC3 microglia, including reduced LPS-induced IL-6, reduced IL-8 by free urolithins, reduced MyD88 signaling with Uro-A, and inhibition of NF-κB nuclear translocation by several free and conjugated forms (19). Johnson et al. broadened this neuroimmune evidence beyond flavanol- and urolithin-derived metabolites by showing that enterolactone and equol were BBB-permeable in PAMPA assays and that genistein reduced LPS-induced nitric oxide and IL-6 in BV-2 microglia (25).

The fourth layer is pathology-state validation, because metabolite effects may differ between chronic aging, vascular cognitive impairment, and acute BBB injury (26, 27). In experimental traumatic brain injury, Gong et al. reported that urolithin A reduced brain edema and BBB permeability, increased tight-junction proteins, and attenuated Akt/mTOR and IKKα/NF-κBsignaling (26). In subarachnoid hemorrhage, Liu et al. showed that urolithin A improved neurological deficits, BBB disruption and cerebral edema at 24 h, with protection linked to AMPK/mTOR-dependent autophagy (27). These acute injury models should not be treated as direct surrogates for brain aging, but they support the broader concept that MPMs can influence BBB integrity, neuroinflammation, edema biology and stress-response pathways when the NVU is pathologically challenged (26, 27). Overall, the mechanistic literature supports a cautious metabolite–barrier–response model in which BBB access is necessary but insufficient, and neurovascular relevance depends on coupled evidence of barrier preservation, inflammatory restraint and disease-stage-appropriate functional outcomes (Table 2).

**Table 2:** Mechanistic BBB/NVU evidence for MPMs and related low-molecular-weight phenolic catabolites.

Mechanistic domain	Model/study	Metabolite/exposure	BBB/NVU signal
BBB access + neuroimmune modulation	HBMEC BBB model, N9 microglia, neuronal models Figueira et al. (17)	Catechol sulfate, pyrogallol sulfate and related phenolic sulfates; 5 μM, based on reported plasma range 0.3–12 μM	Phenolic sulfates crossed the BBB model; pyrogallol sulfate reduced LPS-induced microglial TNF-*α* by ~2-fold and modulated NF-κB/IκBα signaling.
Flavan-3-ol catabolite brain exposure	in silico, HBMEC BBB, rat and pig models Angelino et al. (24)	5-(hydroxyphenyl)-γ-valerolactonesulfate; 5 μM, 2 h	Detected across BBB compartments; reported upper/lower chamber levels 0.42 ± 0.03 μM and 0.05 ± 0.01 μM; sulfate PVL detected in rat and pig brain cortex after dietary exposure.
Barrier phenotype beyond permeability	HBMEC BBB transport and Wistar rat tissue distribution Carecho et al. (18)	Pyrogallol sulfate, phloroglucinol sulfate, resorcinol sulfate	Pyrogallol sulfate showed high BBB permeability, increased *β*-catenin membrane expression, reduced ZO-1 membrane gaps, and reached rat brain rapidly.
Urolithin-conjugate BBB protection	HBMEC BBB model and HMC3 human microglia Garay-Mayol et al. (19)	Urolithin A, urolithin B, isourolithin A and glucuronide/sulfate conjugates; 5 μM	Urolithins/conjugates crossed the BBB model; Uro-B/Uro-B sulfate showed high transport; Uro-A reduced Na-F permeability; selected metabolites rescued TEER; urolithins reduced LPS-induced IL-6/IL-8 and NF-κB nuclear translocation.
Evidence beyond flavanol/urolithin metabolites	PAMPA-BBB and BV-2 microglia Johnson et al. (25)	Enterolactone, equol, genistein, daidzein; 10–20 μM	Enterolactone and equol showed high BBB passive permeability; genistein reduced LPS-induced NO by 68% and IL-6 by 80.4% at 20 μM; enterolactone reduced NO, IL-6 and TNF-α.
In vivo BBB injury validation	experimental TBI mouse model Gong et al. (26)	Urolithin A; 2.5 mg/kg	Reduced brain edema and BBB permeability, increased tight-junction proteins, and attenuated Akt/mTOR and IKKα/NF-κBsignaling.
Pathology-stage relevance	SAH rat model Liu et al. (27)	Urolithin A; acute injury paradigm	Improved neurological deficits, BBB disruption and cerebral edema at 24 h; protection involved AMPK/mTOR-dependent autophagy.

Most mechanistic studies use simplified BBB systems or acute injury models; therefore, BBB transport and barrier rescue should be interpreted as mechanistic plausibility rather than proof of durable neurovascular protection in human brain aging. BBB, blood–brain barrier; HBMEC, human brain microvascular endothelial cell; IL, interleukin; LPS, lipopolysaccharide; Na-F, sodium fluorescein; NF-κB, nuclear factor kappa B; NVU, neurovascular unit; PVL, phenyl-γ-valerolactone; SAH, subarachnoid hemorrhage; TBI, traumatic brain injury; TEER, transendothelial electrical resistance; TNF-α, tumor necrosis factor-alpha; Uro, urolithin; ZO-1, zonula occludens-1.

Several methodological limitations temper the mechanistic evidence. Many BBB studies rely on simplified *in vitro* endothelial models that cannot fully reproduce the cellular complexity, shear stress, transporter dynamics, pericyte interactions, astrocytic end feet, and immune signaling of the human neurovascular unit. Experimental concentrations, although often selected to approximate reported circulating ranges, may not reflect chronic dietary exposure across diverse individuals. In addition, traumatic brain injury and subarachnoid hemorrhage models demonstrate BBB protection under acute injury conditions, but these paradigms should not be treated as direct surrogates for gradual physiological brain aging, cerebral small-vessel disease, or mixed Alzheimer’s–vascular pathology. Thus, BBB transport and barrier rescue should be interpreted as mechanistic plausibility rather than proof of durable neurovascular protection in humans.

## 6 Pathology-informed precision nutrition framework

Future polyphenol–gut–brain studies could operationalize neuroprotection as a multilevel phenotype linking diet, microbial biotransformation, circulating metabolite exposure, BBB/NVU integrity, and brain-aging outcomes, rather than as cognitive change inferred from dietary intake alone (1, 3, 4, 15). At the exposure level, dietary assessment should be paired with urinary MPM scores and targeted LC–MS/MS quantification of plasma phenolic acids, phenyl-*γ*-valerolactones, urolithins, enterolignans, and sulfate/glucuronide conjugates, because urine captures cumulative microbial-metabolic signatures whereas plasma or serum better reflects the BBB-facing metabolite pool (12, 15, 20). CSF profiling is not feasible for routine nutrition trials, but in selected clinical or translational cohorts it can provide complementary evidence of brain-adjacent metabolite exposure (15).

A gut-barrier layer should be added to this workflow. In human studies, intestinal permeability can be assessed using serum zonulin, lactulose/mannitol or multi-sugar permeability tests, circulating intestinal fatty-acid binding protein, LBP/sCD14, endotoxin/LPS-related markers, and fecal calprotectin. In experimental models, epithelial TEER, FITC-dextran flux, mucus integrity, occludin, claudin-1, ZO-1, and inflammatory epithelial signaling can be paired with MPM production and BBB/NVU readouts. This would allow future studies to test whether polyphenols increase or reshape systemic MPM exposure by improving intestinal barrier function, altering microbial metabolism, or both.

Implementation of a neurovascular responder metabotype will require careful adjustment for biological and environmental confounders. Age, sex, baseline dietary pattern, recent antibiotic or probiotic exposure, medication use, diabetes, metabolic syndrome, renal and hepatic function, ethnicity, geography, and habitual microbiome composition can all influence MPM production, circulation, and interpretation. Future studies should therefore collect standardized dietary, medication, metabolic, and microbiome metadata alongside urine/plasma metabolomics and BBB/NVU readouts. Clinically, targeted LC–MS/MS profiling is feasible in research and specialized laboratory settings, but cost, analytical standardization, access to validated panels, and interpretation of conjugated versus free metabolites remain barriers to routine use. The most practical early populations for translation include mild cognitive impairment, vascular cognitive impairment, diabetes-associated cognitive decline, cerebral small-vessel disease, and older adults with high cardiometabolic risk.

The microbial layer should include fecal microbiome profiling, metagenomics, metatranscriptomics, and microbial enzyme mapping, because inter-individual variation in phytonutrient biotransformation may determine whether participants become metabolite producers or non-producers (4). Standardized sampling windows, fasting status, targeted LC–MS/MS panels, reporting of conjugated versus free metabolites, and harmonized cognitive/NVU endpoints will be essential for reproducibility across cohorts (4, 12, 13, 15, 20). The vascular pathology layer should measure BBB integrity using claudin-5, occludin, ZO-1, *β*-catenin localization, albumin/IgG leakage and, where feasible, permeability imaging, while experimental models should report TEER and Na-F permeability to link transport with barrier phenotype (1, 3, 18, 19). Endothelial activation should be assessed through ICAM-1, VCAM-1, eNOS, ROS, and oxidative injury markers, while pericyte and basement-membrane integrity should be captured through PDGFRβ, pericyte coverage, and matrix remodeling (1, 3).

Astrocytic and microglial responses should include GFAP, AQP4 polarization, Iba1, CD68, TREM2, IL-1β, TNF-*α*, IL-6, NLRP3, and NF-κB, because MPMs may act through endothelial–glial inflammatory circuits rather than direct neuronal antioxidant mechanisms alone (3, 17–19). Brain-aging outcomes should then be aligned with neurovascular vulnerability by measuring white matter integrity, cerebral blood flow or cerebral blood volume, executive function, frontal cognition, memory, and processing speed (20, 21, 23). This framework could allowaneurovascular responder metabotype to be tested prospectively: an individual whose microbiome-generated phenolic metabolite profile is associated with measurable BBB/NVU preservation and cognitive resilience (4, 13, 20, 21). Such a framework would allow nutritional neuroprotection to be evaluated as preservation of tissue-level neurovascular integrity, not only as improvement in cognitive test scores.

## 7 Discussion: a metabolite–barrier–response model for brain aging

This Mini Review reframes polyphenol neuroprotection as a metabolite–barrier–response problem rather than a parent-compound intake problem (1, 3, 4). Parent polyphenols remain important dietary precursors, but the biologically relevant exposure for brain aging is more plausibly the circulating pool of MPMs generated by gut biotransformation (3, 4, 12, 13). This shift may be useful because BBB disruption, endothelial junctional changes, pericyte and astrocyte dysfunction, and inflammatory glial signaling are increasingly recognized as central features of aging and neurodegeneration (1, 4). The sequence-polyphenol-rich diet, microbial conversion, measured metabolite exposure, BBB/NVU response, and cognitive phenotypeoffers a testable alternative to broad antioxidant claims (3, 4, 15).

Human evidence is consistent with first half of this sequence by showing that MPMs can be measured as exposure biomarkers and may relate to cognition (12, 13, 15, 20). Urinary MPMs have been associated with better frontal-lobe performance in older adults, supporting their role as candidate neurocognitive exposure biomarkers (20). Plasma and CSF profiling in individuals at risk of dementia strengthens biological plausibility by showing that multiple diet-derived metabolites are detectable in brain-adjacent fluid compartments (15). Biomarker studies show that urinary *γ*-valerolactone metabolites and phenyl-γ-valerolactones can track flavan-3-ol exposure more objectively than dietary questionnaires alone (12, 13). Mechanistic studies support the second half of the model by linking BBB transport with barrier modulation through *β*-catenin, ZO-1, TEER and Na-F permeability, and with inflammatory pathways involving NF-κB, TNF-*α*, IL-6 and MyD88 (17–19, 24).

However, causality between MPMs and durable neurovascular protection in human brain aging is not established (15, 20, 23). Most studies do not yet measure diet, microbial enzyme capacity, urine and plasma metabolites, BBB/NVU biomarkers, neuroimaging and cognition within the same design (12, 13, 15, 20, 23). Some *in vitro* studies use simplified BBB models or metabolite concentrations that may not fully reproduce chronic human exposure, even when designed around reported circulating ranges (17–19, 24). Acute injury models support BBB-protective potential, but traumatic brain injury and subarachnoid hemorrhage cannot be directly equated with aging, cerebral small-vessel disease or mixed Alzheimer’s–vascular pathology (26, 27).

### 7.1 Limitations, unresolved controversies, and future directions

Several unresolved controversies now define the field. First, urine, plasma/serum, and CSF do not represent the same exposure construct: urine indexes cumulative microbial-metabolic output, plasma/serum represents the immediate BBB-facing pool, and CSF provides a selected brain-adjacent compartment. Second, MPM exposure is not determined by microbial production alone; intestinal permeability, epithelial uptake, hepatic conjugation, renal clearance, and inflammatory tone may all modify systemic availability. Third, BBB crossing should not be conflated with neurovascular benefit. A metabolite may cross a simplified barrier model yet fail to preserve tight-junction organization, reduce endothelial activation, alter glial inflammatory signaling, or improve cognition in chronic aging. Fourth, transporter mechanisms remain insufficiently resolved, particularly for distinguishing phenolic acids, phenyl-*γ*-valerolactones, and urolithin conjugates. Finally, acute injury models are useful for barrier-stress biology but are imperfect proxies for gradual physiological aging, cerebral small-vessel disease, or mixed Alzheimer’s–vascular pathology.

Prospective studies should therefore pre-specify MPM panels, use human-relevant concentrations and time courses, stratify participants by urolithin, valerolactone, or enterolignan metabotype, and combine dietary data, fecal metagenomics or enzyme mapping, intestinal permeability markers, urine/plasma MPMs, transporter-resolved BBB assays or permeability imaging, white-matter and cerebral blood-flow measures, and frontal/executive cognitive outcomes. This design would allow the field to test whether MPMs are causal neurovascular effectors, exposure biomarkers, or both (4, 13, 20). This framework is particularly relevant to metabolic syndrome, diabetes-associated brain aging, cerebral small-vessel disease, and vascular contributions to Alzheimer’s disease, where BBB dysfunction, endothelial inflammation, and impaired cerebral perfusion overlap with dietary and microbiome variability (1, 4, 23, 32). Trials should measure urinary MPM signatures, plasma BBB-facing metabolites, fecal microbial enzyme capacity, intestinal permeability markers, BBB transporter expression or activity where feasible, permeability imaging, cerebral blood flow, white matter integrity, endothelial markers and frontal/executive cognition, particularly because BBB breakdown has been proposed as an early biomarker of human cognitive dysfunction (1, 4, 15, 33). An immediately testable prediction is that that older adults with favorable microbial conversion capacity, preserved or improved intestinal barrier function, and higher plasma BBB-facing MPM profiles will show lower BBB permeability, better white-matter integrity, and stronger frontal/executive performance after adjustment for diet quality, renal and hepatic function, cardiometabolic risk, and baseline cognitive status. Moving from food-frequency associations to metabolite-resolved, microbiome-informed, neurovascularly anchored trials may explain heterogeneous responses to polyphenol-rich diets and provide a more rigorous framework for diet-derived neuroprotection in aging (3, 4, 23).

## Glossary

AD: Alzheimer’s disease
AQP4: Aquaporin-4
BBB: Blood–brain barrier
CBV: Cerebral blood volume
CD68: Cluster of differentiation 68
CSF: Cerebrospinal fluid
CTT: Color Trail Test
eNOS: Endothelial nitric oxide synthase
EVOO: Extra-virgin olive oil
GFAP: Glial fibrillary acidic protein
HBMEC: Human brain microvascular endothelial cell
HMC3: Human microglial cell line
ICAM-1: Intercellular adhesion molecule-1
IL: Interleukin
LC–MS/MS: Liquid chromatography–tandem mass spectrometry
LPS: Lipopolysaccharide
MCI: Mild cognitive impairment
MPM: Microbial phenolic metabolite
MyD88: Myeloid differentiation primary response 88
Na-F: Sodium fluorescein
NF-κB: Nuclear factor kappa B
NLRP3: Nucleotide-binding oligomerization domain-like receptor family pyrin domain-containing 3
NVU: Neurovascular unit
PDGFRβ: Platelet-derived growth factor receptor beta
PVL: Phenyl-*γ*-valerolactone
ROS: Reactive oxygen species
SAH: Subarachnoid hemorrhage
TBI: Traumatic brain injury
TEER: Transendothelial electrical resistance
TNF-*α*: Tumor necrosis factor-alpha
UBG: Urolithin B glucuronide
Uro-A: Urolithin A
VAG: Vanillic acid glucuronide
VCAM-1: Vascular cell adhesion molecule-1
ZO-1: Zonula occludens-1
